# First-pass isolation and procedural metrics associated with catheter ablation outcomes for atrial fibrillation: a retrospective cohort study

**DOI:** 10.3389/fcvm.2026.1900303

**Published:** 2026-08-25

**Authors:** Zexi Li, Fanghui Li, Xianjin Hu, Aobo Gong, Ying Cao, Bangjiaxin Ren, Wenjie Li, Yifan Zhou, Rui Zeng

**Affiliations:** Department of Cardiology, West China Hospital, Sichuan University, Chengdu, Sichuan, China

**Keywords:** arrhythmia recurrence, atrial fibrillation, first-pass isolation, pulmonary vein isolation, radiofrequency catheter ablation

## Abstract

**Background:**

First-pass isolation (FPI) during pulmonary vein isolation (PVI) is an acute procedural endpoint that may reflect the quality of initial lesion delivery and has been proposed as a predictor of durable ablation success. This study aimed to evaluate the association between FPI and post-ablation outcomes, identify factors associated with FPI achievement, and determine whether procedural metrics add prognostic information beyond conventional clinical variables in atrial fibrillation (AF) patients.

**Methods:**

This retrospective cohort study included 313 AF patients undergoing first-time radiofrequency catheter ablation between August 2023 and January 2025. Patients were classified into two groups: those without FPI in either ipsilateral PVs (NEITHER, *n* = 60) and those with FPI in at least one of the two ipsilateral PVs (IFPI, *n* = 253). For additional analyses, the IFPI group was further classified into patients with FPI in both ipsilateral PVs (BOTH, *n* = 151) and those with FPI in either ipsilateral PVs (EITHER, *n* = 102). Procedural metrics for each PV circle, including C3-FOT ratio and gap number, were assessed using the AIFV system (an analytic platform analyzing raw CARTO3 data). The clinical endpoint was freedom from documented atrial arrhythmias after the blanking period, including AF, atrial tachycardia, or atrial flutter lasting longer than 30 s during follow-up.

**Results:**

FPI was achieved in 64.5% (404/626) of ipsilateral PV circles. Higher C3-FOT ratio was independently associated with greater FPI achievement, whereas right-sided PV location and higher gap number were associated with reduced FPI achievement. During a median follow-up of 21.1 months, 81 patients (25.9%) experienced recurrence. The NEITHER group had lower recurrence-free survival than the IFPI group (log-rank *P* = 0.013) and a higher adjusted recurrence risk (HR: 1.680; 95% CI: 1.021–2.763; *P* = 0.041). Failure to achieve C3-FOT qualification in both PV sides and total gap burden >2 were also independently associated with recurrence. C3-FOT qualification appeared to provide more prognostic information than FPI beyond traditional clinical factors.

**Conclusions:**

IFPI was independently associated with a lower risk of atrial arrhythmia recurrence, while FPI achievement was related to favorable procedural characteristics including lower gap burden and higher C3-FOT ratio.

## Introduction

Atrial fibrillation (AF) is the most common sustained cardiac arrhythmia in clinical practice and represents a major source of cardiovascular morbidity and healthcare burden worldwide. AF is associated with impaired quality of life and an elevated risk of adverse outcomes, including stroke and heart failure (1). With population ageing, the burden of AF continues to increase (2), highlighting the need for more effective and durable rhythm-control strategies. Catheter ablation has become an established treatment option for symptomatic AF, and pulmonary vein isolation (PVI) remains the cornerstone lesion set in contemporary AF ablation (3). Nonetheless, recurrence after ablation continues to limit long-term efficacy (4), underscoring the need to better characterize procedural quality and to identify intraoperative markers associated with subsequent clinical outcomes.

In this setting, first-pass isolation (FPI) during PVI refers to successful isolation of the pulmonary veins (PVs) from the surrounding left atrium following the initial encirclement, without the requirement for additional touch-up ablation (5). Failure to achieve FPI may result from incomplete transmural conduction block in one or more segments of the lesion set. Thus, FPI may represent a binary acute procedural endpoint that reflects the combined effects of lesion contiguity, energy delivery, catheter–tissue contact, and anatomical or tissue-related factors. Prior studies have suggested that FPI is associated with improved long-term freedom from recurrence (6–9) and a lower rate of PV reconnection during re-do procedures (6, 10). Accordingly, a better understanding of the factors associated with FPI may provide important insight into how to optimize the efficacy and efficiency of AF ablation. In a multicenter prospective registry analysis, Kreidieh et al. reported that operator experience, patient comorbidities, and procedural strategy may influence FPI achievement (5). However, the factors associated with FPI have not been fully elucidated, and the current evidence for its value in predicting long-term procedural success remains limited.

Although long-term freedom from recurrence is the ultimate marker of ablation success, FPI represents a valuable acute procedural endpoint. Existing models for predicting recurrence have largely relied on baseline clinical variables or composite risk scores, with variable performance across studies (11, 12). This may partly reflect that post-ablation outcome is influenced not only by baseline patient factors, but also by lesion delivery and the overall quality of PVI. By focusing on an acute intraprocedural endpoint like FPI, it may be possible to gain a clearer perspective on procedural quality, thereby informing strategies to optimize ablation and improve outcomes. Thus, this study aimed to (1) evaluate the association between FPI status and clinical outcomes in patients with AF undergoing radiofrequency catheter ablation (RFCA), (2) identify clinical and procedural factors associated with FPI during PVI, and (3) assess whether key procedural metrics provide prognostic information beyond conventional clinical risk factors.

## Methods

### Study design and population

**Figure 1:**
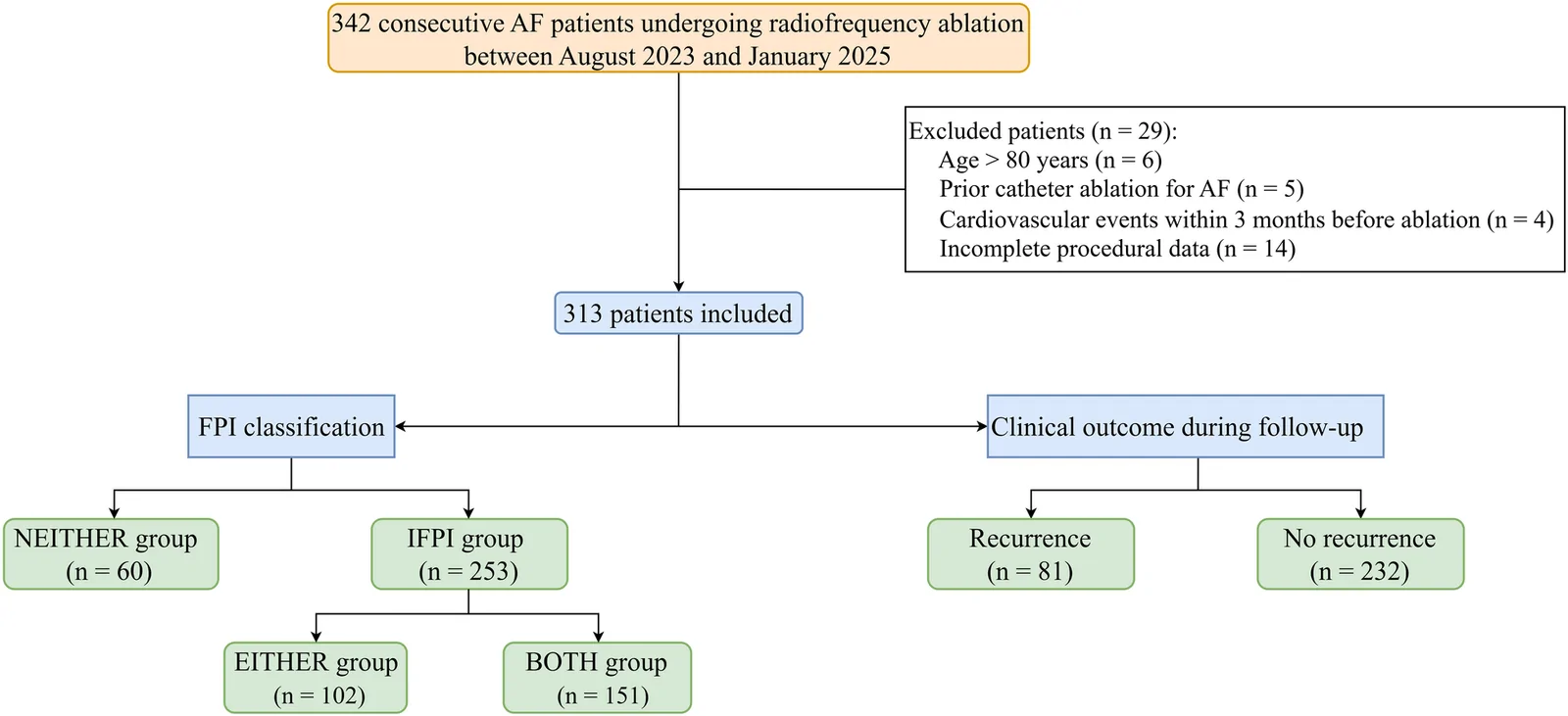
Study flow diagram.

This retrospective cohort study consecutively enrolled 342 patients with AF who underwent RFCA at West China Hospital of Sichuan University between August 2023 and January 2025. The inclusion criteria were as follows: (1) individuals aged 18–80 years; (2) patients with a diagnosis of paroxysmal or persistent AF, confirmed via 12-lead electrocardiogram, Holter monitoring, remote monitoring, or implantable device recordings within the 6 months before the ablation. The exclusion criteria were as follows: (1) individuals with a history of prior catheter ablation; (2) individuals who had suffered cardiovascular events, such as acute myocardial infarction or percutaneous coronary intervention, within the 3 months preceding the ablation procedure; (3) individuals with incomplete procedural data. Ultimately, 313 patients were retained for the final analysis (Figure 1).

The patients' baseline characteristics were extracted from their electronic medical records. These included (1) demographic data: age, sex, body mass index (BMI), smoking status, and alcohol consumption; (2) AF-related information: AF type, AF duration, antiarrhythmic drug history, CHA2DS2-VASc score, HAS-BLED score; (3) comorbidities; (4) echocardiographic data: left atrial diameter (LAD), left ventricular diameter (LVD) and left ventricular ejection fraction (LVEF); (5) laboratory data: hemoglobin (Hb), creatinine (Cre), fibrinogen (FIB) and pro-brain natriuretic peptide (proBNP).

The study complied with the principles of the Declaration of Helsinki and was approved by the Ethics Committee of West China Hospital, Sichuan University (Approval No. 2022-323). The requirement for informed consent was waived due to the retrospective nature of the study.

### RFCA procedure

Catheter ablation was conducted under local anesthesia with lidocaine and under continuous administration of direct oral anticoagulants or warfarin (INR 2-3). Following successful percutaneous femoral venous access and transseptal puncture (VIZIGO™ Bi-directional Guiding Sheath, Biosense Webster, Inc.), systemic heparin was administered to sustain an activated clotting time of 250–350 s. Subsequently, a PentaRay electrode catheter (Biosense Webster, Inc.) was strategically placed within the left atrium to develop a three-dimensional map via the CARTO3 system (Biosense Webster, Inc.). The ablation protocol adhered to a uniform, standardized method. Specifically, an ablation index (AI)-guided circumferential PVI (>10 mm outside the PV ostia) was routinely performed at 50 W with a target contact force of 5–20 g, utilizing a ThermoCool SmartTouch SF catheter (Biosense Webster, Inc.). Radiofrequency (RF) energy was delivered point by point to form a continuous circle. The target AI was 450–500 for anterior segments, 400–450 for roof segments, and 350–400 for bottom and posterior segments. FPI was defined as successful PVI achieved in the initial encirclement of the ipsilateral PVs, as confirmed by monitoring of their electrical activity. If initial encirclement failed to achieve isolation, touch-up ablation was performed to ensure the completion of PVI. Following PVI, additional linear ablation was performed as deemed necessary at the discretion of the operating electrophysiologist. The absence of PV potentials, detected by the PentaRay catheter, was established as the ablation endpoint. If AF persisted after ablation, a 200J synchronous electrical cardioversion would be administered to restore sinus rhythm.

### AIFV-based procedural assessment

Ablation lesion tags were recorded using the VisiTag module. Post-procedural quantitative assessment was performed using the AIFV system, an analytic platform that extracts and analyzes raw data from the CARTO3 system to assess the quality of ablation across the bilateral PVs. The AIFV system generates detailed parameters for each PV circle and provides a comprehensive analytical report on procedural ablation characteristics. This system was also applied in a previous study and showed good feasibility (13, 14). The parameters provided by the AIFV system in our center were defined as follows: (1) C3-FOT ratio: the proportion of lesions within the PV circle with contact force > 4 g for more than 30% of the ablation duration. (2) C3-FOT qualified: a C3-FOT ratio ≥75%. When unqualified, the C3-FOT deficiency location was defined as the area containing lesions not meeting the C3-FOT criterion. (3) Gap: an inter-lesion distance (ILD) ≥6 mm. (4) Big gap: any gap >8 mm. (5) Break point ratio: the proportion of break points among all ablation lesion points within the PV circle, with break points defined as either any 4 lesions with pairwise ILD ≤ 3 mm or at least 6 lesions located within a radius of 6 mm. (6) SURPOINT (Top4) ratio: the proportion of lesions within the PV circle classified into the top four AI categories, using 50-unit intervals for categorization (e.g., 450–500 as one category). (7) Total procedure time: the duration from the initial to the final RF delivery throughout the entire ablation procedure. (8) Total PVI time: the duration from the initial to the final RF delivery within the PV circle. (9) RF time: the cumulative duration of RF delivery within the PV circle. (10) RF time ratio: the proportion of RF time relative to total PVI time. (11) Ablation lesion points: the number of lesions within the PV circle.

Scoring was performed for each PV circle and the entire ablation process based on specific parameters, as detailed in Supplementary File S1. For detailed analysis, each PV circle was segmented into four distinct regions: the roof, anterior, posterior, and bottom sections (Supplementary File S2). Clinical characteristics, intraoperative data, and perioperative complications were systematically documented and subjected to subsequent offline analysis. The electrophysiologists remained blinded to the results of the research analysis and evaluations.

### Follow-up and endpoints

During the 3-month blanking period, oral antiarrhythmic drugs and anticoagulation therapy were prescribed. Patients underwent scheduled follow-up with 24-h Holter monitoring at 1, 3, 6, and 12 months after ablation. A 12-lead surface electrocardiogram was performed whenever patients reported arrhythmic symptoms. Follow-up thereafter was continued through outpatient visits, telephone calls, or online consultations, with additional rhythm monitoring performed at the discretion of the treating physician or when recurrence was clinically suspected. All included patients completed at least 12 months of follow-up after ablation.

The procedural endpoint was FPI of the ipsilateral PV circle. FPI was selected because it is an immediately observable and clinically interpretable acute electrophysiological endpoint that provides a meaningful basis for patient classification and permits comparison with previous FPI studies. At the patient level, based on previous studies (6, 8), patients were primarily categorized into two groups: those with FPI in at least one ipsilateral PV circle (IFPI group) and those without FPI in either ipsilateral PV circle (NEITHER group). For additional analyses, patients were also classified into three groups: those with FPI in both ipsilateral PV circles (BOTH group), those with FPI in either ipsilateral PV circle (EITHER group), and the NEITHER group. The clinical endpoint was freedom from documented atrial arrhythmias after the blanking period, including AF, atrial tachycardia, or atrial flutter lasting longer than 30 s during follow-up.

### Statistical analysis

Since all continuous variables were non-normally distributed, they were presented as median [interquartile range (IQR)] and were compared using Wilcoxon rank-sum tests. Categorical variables were presented as counts (percentages) and compared using chi-square or Fisher's exact tests. Univariable and multivariable mixed-effects logistic regression models with patient-level random intercepts were applied to identify factors independently associated with FPI. Candidate variables were selected based on clinical relevance, prior literature, and collinearity considerations. Restricted cubic spline (RCS) analyses were performed to investigate the dose-response relationship with FPI, with likelihood ratio tests applied to identify any potential nonlinearity.

Recurrence-free survival was estimated using the Kaplan–Meier analysis and compared with the log-rank test. Cox proportional hazards models were constructed to evaluate the associations of FPI status and other procedural metrics with atrial arrhythmia recurrence. Covariates included in the adjusted Cox models were prespecified according to clinical relevance, potential confounding, and model parsimony. Subgroup analyses were performed using the binary FPI classification, and interactions between binary FPI status and stratification variables were assessed using likelihood ratio tests.

To evaluate whether procedural metrics provided incremental prognostic information, a separate baseline clinical model was constructed using conventional clinical variables, including age, sex, BMI, AF type, AF duration, LAD, heart failure, and LVEF (11, 15, 16). Incremental model performance was assessed by Harrell's C-index for recurrence during follow-up. In Supplementary analyses for 1-year recurrence, discrimination and reclassification were further evaluated using the area under the curve (AUC), net reclassification improvement (NRI), and integrated discrimination improvement (IDI). All analyses were performed using SPSS software (version 27.0, IBM Corporation, United States) and R software (version 4.4.3, R Foundation for Statistical Computing, Austria). A two-sided *P* < 0.05 was considered statistically significant.

## Results

### Study population and clinical outcomes

A total of 313 AF patients were included in the analysis. According to FPI status, 253 patients were assigned to the IFPI group, including 151 in the BOTH group and 102 in the EITHER group, whereas 60 patients were assigned to the NEITHER group. After a median follow-up of 21.1 (14.8–23.6) months, 81 (25.9%) patients experienced atrial arrhythmia recurrence after RFCA (Figure 1), with a median time to recurrence of 6.0 (3.8–10.5) months. The 1-year recurrence rate was 22.0% (69/313). Repeat ablation was performed in 20 patients (6.4%). Compared with patients without recurrence, those with recurrence had a lower proportion of paroxysmal AF, a higher prevalence of heart failure, longer AF duration, larger LAD and LVD, lower LVEF, and higher proBNP levels (all *P* < 0.05; Table 1). The NEITHER group had a significantly higher BMI than the IFPI group (*P* = 0.029), with no significant differences in other baseline characteristics (Supplementary File S3).

**Table 1:** Baseline and procedural characteristics of the study population.

Characteristics	Total (*n* = 313)	Non-recurrence (*n* = 232)	Recurrence (*n* = 81)	*P* value
Demographic and clinical characteristics				
Age, years	62.0 (55.0−70.0)	61.5 (55.0–70.0)	62.0 (55.0–68.0)	0.736
Male, *n* (%)	183 (58.5)	137 (59.1)	46 (56.8)	0.722
BMI	24.9 (22.6–27.0)	24.6 (22.6–26.6)	25.3 (22.4–28.0)	0.242
Smoke, *n* (%)	94 (30.0)	65 (28.0)	29 (35.8)	0.188
Drink, *n* (%)	90 (28.8)	61 (26.3)	29 (35.8)	0.104
Paroxysmal AF, *n* (%)	188 (60.1)	155 (66.8)	33 (40.7)	**<0.001**
Presence of other arrhythmias, *n* (%)	78 (24.9)	61 (26.3)	17 (21.0)	0.342
Antiarrhythmic drug, *n* (%)	128 (40.9)	91 (39.2)	37 (45.7)	0.309
CHA2DS2-VASc score	2.0 (1.0–3.0)	2.0 (1.0–3.0)	2.0 (1.0–3.0)	0.658
HAS-BLED score	1.0 (0.0–2.0)	1.0 (0.0–2.0)	1.0 (0.5–2.0)	0.223
AF duration, months	12.0 (3.0–25.0)	8.0 (2.0–24.0)	15.0 (5.5–36.0)	**0**.**001**
Comorbidities, *n* (%)				
Heart failure	43 (13.7)	26 (11.2)	17 (21.0)	**0**.**028**
Hypertension	133 (42.5)	99 (42.7)	34 (42.0)	0.913
Diabetes mellitus	50 (16.0)	37 (15.9)	13 (16.0)	0.983
CAD	42 (13.4)	31 (13.4)	11 (13.6)	0.960
History of stroke	33 (10.5)	24 (10.3)	9 (11.1)	0.847
Thyroid disease	16 (5.1)	12 (5.2)	4 (4.9)	0.934
COPD	15 (4.8)	8 (3.4)	7 (8.6)	0.060
Echocardiographic data				
LAD, mm	39.0 (36.0–43.0)	38.0 (35.0–41.75)	41.0 (38.0–47.0)	**<0.001**
LVD, mm	48.0 (45.0–51.0)	48.0 (45.0–50.0)	49.0 (45.5–52.0)	**0**.**032**
LVEF, %	64.0 (59.5–69.0)	65.0 (61.0–70.0)	62.0 (55.5–67.0)	**0**.**001**
Laboratory data				
Hb, g/L	140.0 (132.0–152.0)	142.0 (132.0–153.0)	138.0 (131.5–149.5)	0.208
Cre, umol/L	80.0 (68.0–92.0)	79.5 (68.0–92.0)	80.0 (66.5–89.0)	0.942
FIB, g/L	2.61 (2.29–3.06)	2.65 (2.33–3.08)	2.57 (2.25–3.06)	0.368
proBNP, pg/mL	373.0 (82.5–903.0)	274.5 (72.5–734.5)	578.0 (275.5–1,169.5)	**<0.001**
Procedural details				
PVI plus, *n* (%)	103 (32.9)	67 (28.9)	36 (44.4)	**0**.**010**
Total procedure time, min	62.6 (48.1–82.1)	60.8 (47.6–80.0)	70.1 (49.1–86.7)	**0**.**045**
Gap of PVI circle	1.0 (0.0–2.0)	1.0 (0.0–1.0)	1.0 (0.0–3.0)	**0**.**004**
Big gap, *n* (%)	34 (10.9)	20 (8.6)	14 (17.3)	**0**.**031**
C3-FOT qualified in both PV sides, *n* (%)	231 (73.8)	184 (79.3)	47 (58.0)	**<0.001**
Total VisiTag score, *n* (%)				
V2	4 (1.3)	0 (0)	4 (4.9)	**<0.001**
V3	53 (16.9)	33 (14.2)	20 (24.7)	
V4	88 (28.1)	63 (27.2)	25 (30.9)	
V5	136 (43.5)	113 (48.7)	23 (28.4)	
V6	32 (10.2)	23 (9.9)	9 (11.1)	
FPI status, *n* (%)				
BOTH group	151 (48.2)	120 (51.7)	31 (38.3)	**0**.**003**
EITHER group	102 (32.6)	75 (32.3)	27 (33.3)	
NEITHER group	60 (19.2)	37 (15.9)	23 (28.4)	

AF, atrial fibrillation; FPI, first-pass isolation; CAD, coronary artery disease; COPD, chronic obstructive pulmonary disease; proBNP, pro-brain natriuretic peptide; Cre, creatinine; FIB, fibrinogen; Hb, hemoglobin; LAD, left atrial diameter; LVD, left ventricular diameter; LVEF, left ventricular ejection fraction; PV, pulmonary vein; PVI, pulmonary vein isolation.

Bold *P* values indicate statistical significance (*P* < 0.05).

### Procedural characteristics

Among the 626 ipsilateral PVs from 313 patients, 404 achieved FPI. For non-first-pass PVs, the median touch-up ablation time was 7.0 (3.0–10.0) min. Compared with non-first-pass PVs, first-pass PVs were less frequently right-sided and had more favorable procedural metrics, including a lower break point ratio, fewer gaps, higher C3-FOT and SURPOINT (Top4) ratios. First-pass PVs also required shorter total PVI time and RF time, with fewer ablation lesion points (all *P* < 0.01; Supplementary File S4). The VisiTag score distribution differed significantly between groups, with the highest FPI rate observed in V5 PVs (77.7%), significantly higher than in V2-V4 PVs (*P* < 0.05). At the patient level, patients with recurrence more frequently underwent PVI plus ablation, had longer total procedure time, greater gap burden, a higher prevalence of big gap, and a lower proportion of C3-FOT qualification in both PV sides. The distribution of total VisiTag score also differed significantly (all *P* < 0.05; Table 1). Specifically, patients with a V5 score had the lowest recurrence rate (16.9%), significantly lower than those with V2 or V3 scores (*P* < 0.05). Notably, the proportion of patients undergoing PVI plus ablation did not differ significantly between the IFPI and NEITHER groups (Supplementary File S3).

**Figure 2:**
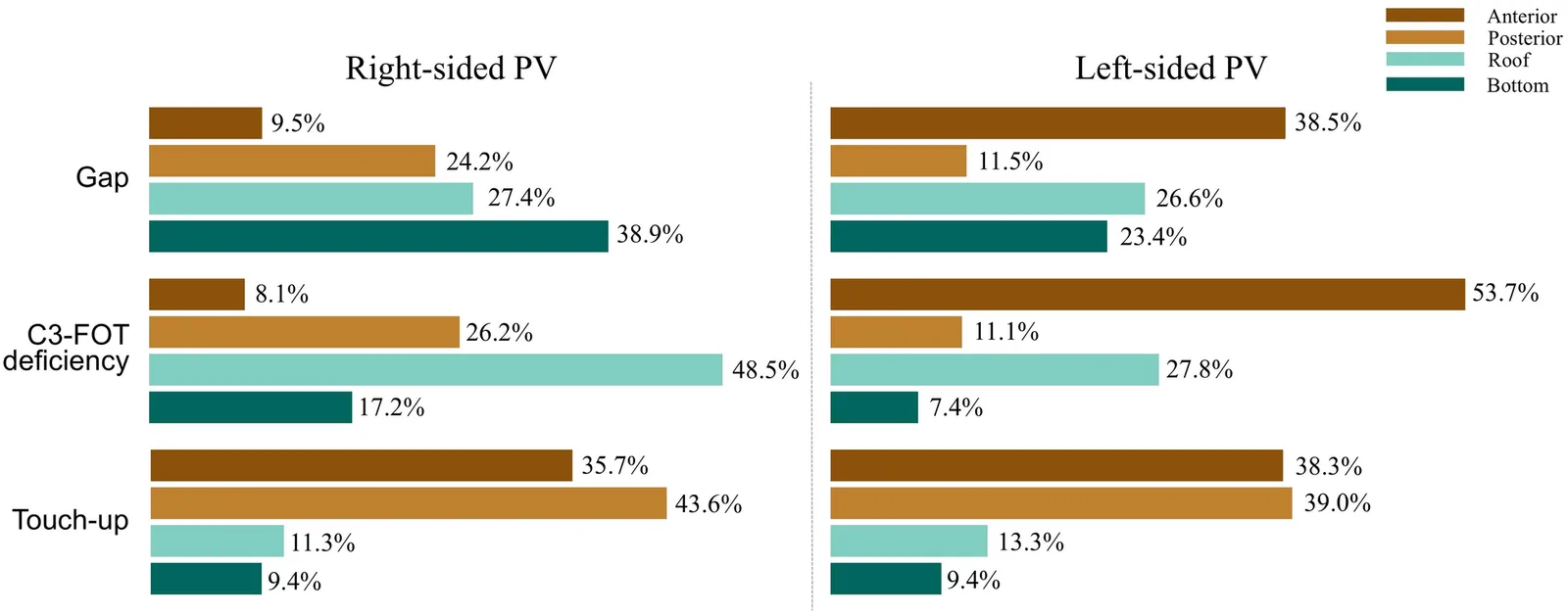
Segmental distribution of gaps, C3-FOT deficiency, and touch-up ablation by PV side.

The segmental distribution of gaps, C3-FOT deficiency, and touch-up ablation is presented in Figure 2. In right-sided PVs, gaps were most frequently located in the bottom (38.9%) and roof segments (27.4%), whereas in left-sided PVs, gaps were more commonly observed in the anterior (38.5%) and roof (26.6%) segments. C3-FOT deficiency was most frequently observed in the roof segment (48.5%) of right-sided PVs and left anterior segment (53.7%) of left-sided PVs. Touch-up ablation was mainly distributed in the anterior (right-sided PVs: 35.7%; left-sided PVs: 38.3%) and posterior (right-sided PVs: 43.6%; left-sided PVs: 39.0%) segments on both sides.

### Variables associated with FPI achievement

In univariable mixed-effects logistic regression analyses, LAD, right-sided PV location, gap number, and break point ratio were negatively associated with FPI achievement, whereas C3-FOT ratio and SURPOINT (Top4) ratio were positively associated with FPI achievement (Table 2). In the multivariable regression model, the inclusion of break point ratio and SURPOINT (Top4) ratio did not improve model fit or materially alter the estimates of the main predictors. Therefore, to maintain model parsimony, the model excluding these two variables was employed for the multivariable analysis. In this model, right-sided PV location (OR: 0.507; 95% CI: 0.307–0.837; *P* = 0.008) and higher gap number (OR: 0.360; 95% CI: 0.255–0.509; *P* < 0.001) were independently associated with reduced FPI achievement, whereas higher C3-FOT ratio was independently associated with increased FPI achievement (OR: 1.148; 95% CI: 1.107–1.192; *P* < 0.001) (Table 2). RCS analysis further supported a linear pattern in the association between C3-FOT ratio and FPI achievement (*P* for nonlinearity = 0.540; Supplementary File S5).

**Table 2:** PV-level mixed-effects logistic regression for FPI.

	Univariate			Multivariate		
	OR	95%CI	*P*	OR	95%CI	*P*
Patient-level variables						
Age, years	1.009	0.988–1.030	0.402	1.024	0.998–1.051	0.073
Sex, male	0.732	0.459–1.169	0.191	0.976	0.547–1.741	0.934
BMI, kg/m^2^	1.000	0.993–1.008	0.918	1.002	0.993–1.011	0.634
Persistent AF	0.787	0.494–1.254	0.314	1.484	0.757–2.908	0.25
LAD, mm	0.938	0.904–0.974	**< 0.001**	0.956	0.905–1.010	0.11
PV-level variables						
Right-sided PV	0.448	0.298–0.672	**<0**.**001**	0.507	0.307–0.837	**0**.**008**
Gap number	0.486	0.384–0.615	**<0**.**001**	0.360	0.255–0.509	**<0**.**001**
C3-FOT ratio, %	1.144	1.107–1.182	**<0**.**001**	1.148	1.107–1.192	**<0**.**001**
Break point ratio, %	0.968	0.946–0.991	**0**.**006**	-	-	-
SURPOINT (Top4) ratio, %	1.071	1.031–1.112	**<0**.**001**	-	-	-

AF, atrial fibrillation; OR, odds ratio; CI, confidence interval; FPI, first-pass isolation; LAD, left atrial diameter; PV, pulmonary vein.

Bold *P* values indicate statistical significance (*P* < 0.05).

### Procedural factors and AF ablation outcomes

**Figure 3:**
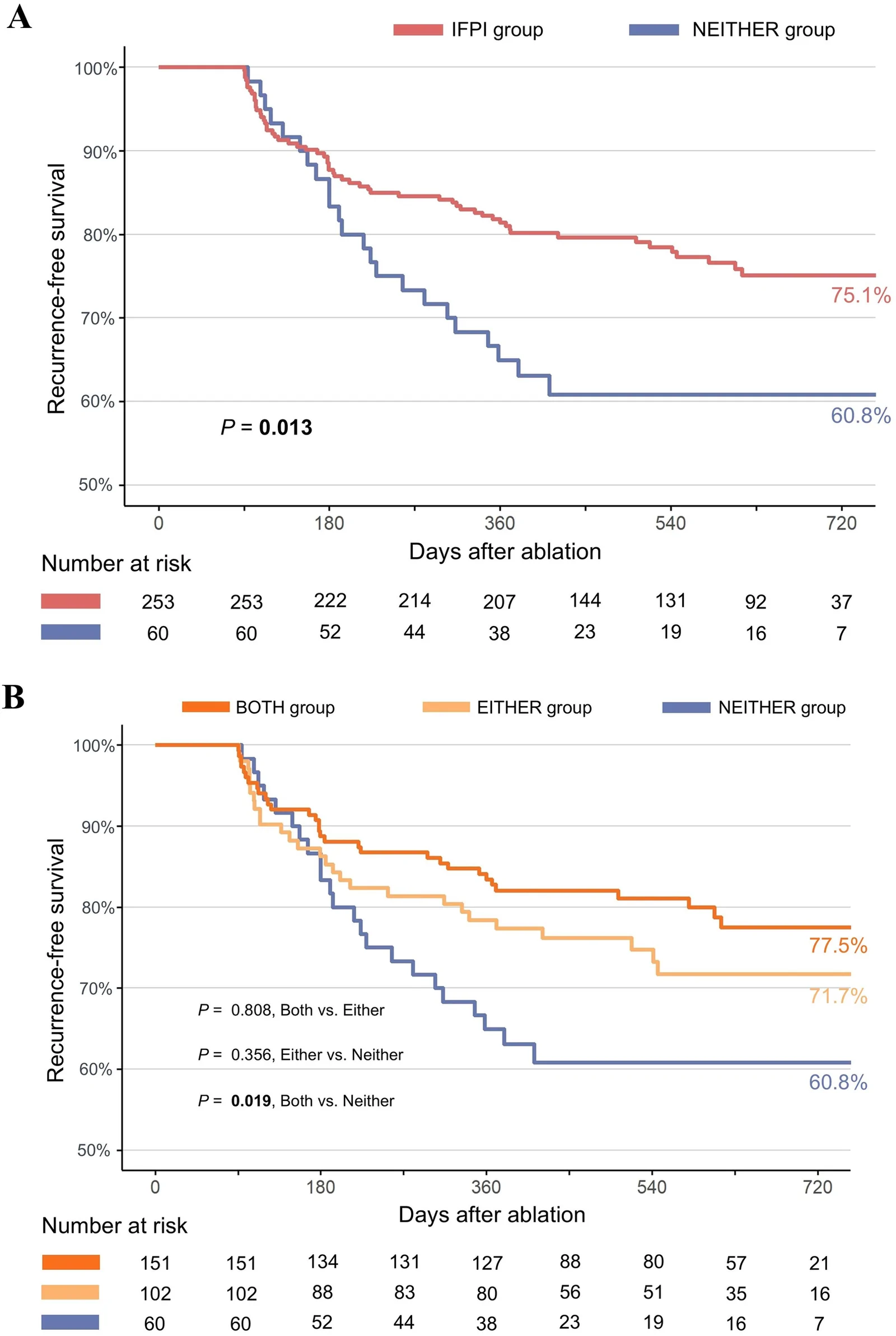
Kaplan–Meier curves for recurrence-free survival by FPI status. **(A)** Binary FPI classification: IFPI vs. NEITHER. **(B)** Three-level FPI classification: BOTH vs. EITHER vs. NEITHER.

The Kaplan–Meier survival analyses showed significantly lower recurrence-free survival in the NEITHER group than in the IFPI group (log-rank *P* = 0.013; Figure 3A), with estimated 720-day recurrence-free survival of 60.8% and 75.1%, respectively. In the three-level analysis, recurrence-free survival showed a graded pattern across the BOTH, EITHER, and NEITHER groups. Pairwise comparison was significant only between the BOTH and NEITHER groups (log-rank *P* = 0.019; Figure 3B).

**Figure 4:**
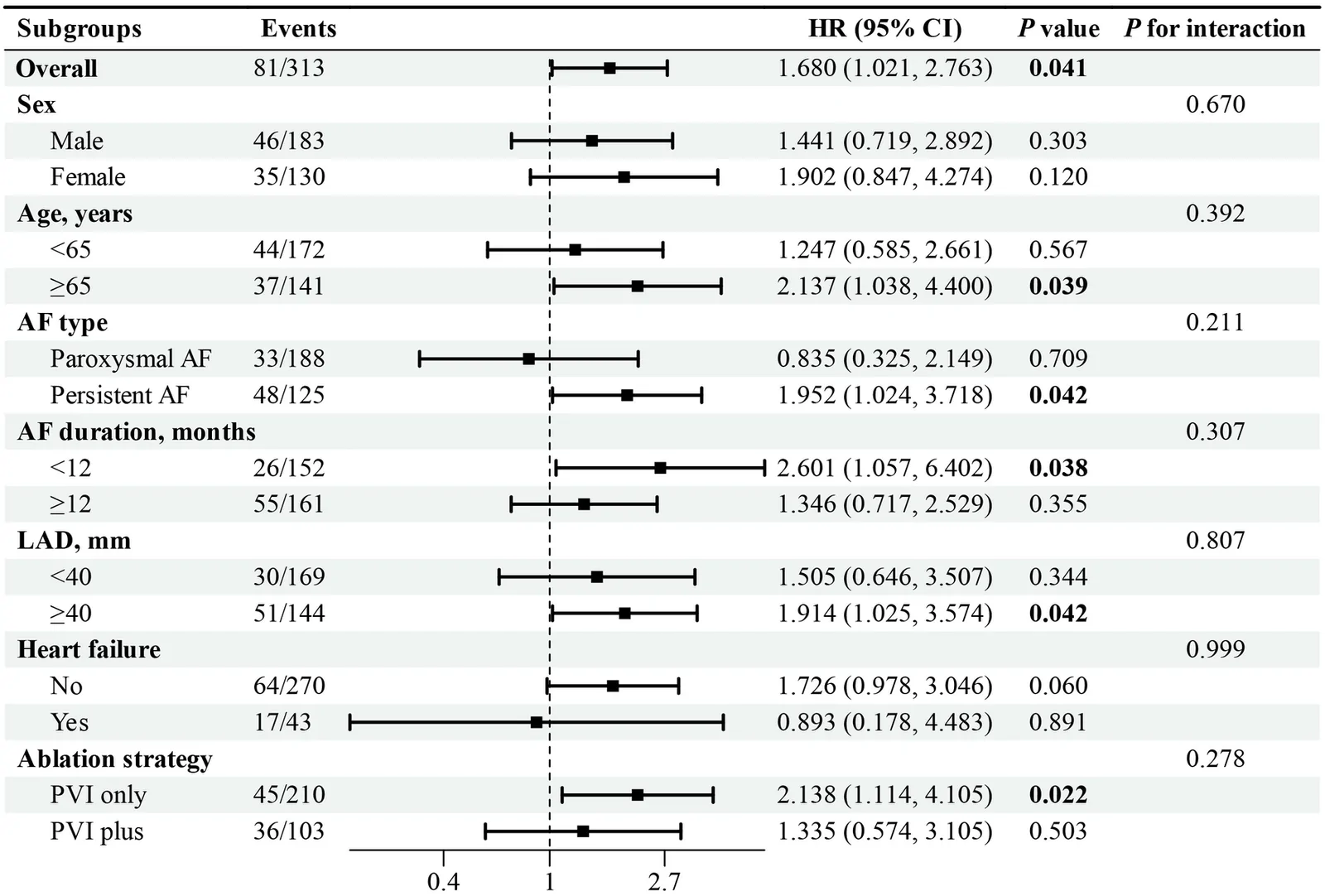
Subgroup analyses of the association between binary FPI status and recurrence.

Cox proportional hazards analyses were performed using both binary and three-level FPI classifications (Table 3). In the binary classification, with the IFPI group as the reference, the NEITHER group was associated with a higher risk of recurrence in both the crude model (HR: 1.835; 95% CI: 1.131–2.977; *P* = 0.014) and the adjusted model (HR: 1.680; 95% CI: 1.021–2.763; *P* = 0.041). In the three-level classification, with the BOTH group as the reference, the NEITHER group remained associated with an increased risk of recurrence in the crude model (HR: 2.081; 95% CI: 1.212–3.572; *P* = 0.008) and the adjusted model (HR: 1.880; 95% CI: 1.074–3.288; *P* = 0.027), whereas the EITHER group was not significantly different from the BOTH group. To further explore whether the association between FPI status and recurrence persisted in different conditions, subgroup analyses were conducted in binary FPI classification. The NEITHER group tended to be associated with a higher recurrence risk in most clinically relevant subgroups. Notably, no significant interactions were observed for sex, age, AF type, AF duration, LAD, heart failure, or ablation strategy (Figure 4).

**Table 3:** Cox proportional hazards models for recurrence according to FPI status.

	Model 1 (Crude)			Model 2 (Adjusted)		
	HR	95%CI	*P*	HR	95%CI	*P*
Binary FPI classification						
IFPI group	Ref.			Ref.		
NEITHER group	1.835	1.131–2.977	**0**.**014**	1.680	1.021–2.763	**0**.**041**
Three-level FPI classification						
BOTH group	Ref.			Ref.		
EITHER group	1.340	0.800–2.245	0.266	1.279	0.753–2.172	0.363
NEITHER group	2.081	1.212–3.572	**0**.**008**	1.880	1.074–3.288	**0**.**027**

FPI, first-pass isolation; HR, hazard ratio; CI, confidence interval; PV, pulmonary vein.

Model 1: unadjusted.

Model 2: adjusted for age, sex, BMI, AF type, AF duration, LAD, PVI plus, heart failure, hypertension, diabetes, CAD, history of stroke.

Bold *P* values indicate statistical significance (*P* < 0.05).

Additional analyses of other procedural metrics are shown in Supplementary File S6. After adjustment, failure to achieve C3-FOT qualification in both ipsilateral PVs (HR: 2.315; 95% CI: 1.456–3.681; *P* < 0.001) and total gap burden >2 (HR: 2.129; 95% CI: 1.286–3.524; *P* = 0.003) were both independently associated with increased recurrence risk. The presence of big gap was not significantly associated with recurrence in either the crude or adjusted model. Compared with V5, total VisiTag scores of V2 and V3 were significantly associated with higher recurrence risk in the crude model, whereas only V2 reached statistical significance after adjustment.

### Prognostic performance analysis

Likelihood ratio tests were used to evaluate whether adding FPI status or other procedural metrics improved the baseline clinical model (Table 4). Adding binary FPI classification only modestly increased the C-index from 0.685 to 0.687, without significant improvement in model fit (ΔLR *χ*^2^ = 3.015; *P* = 0.083). Similarly, three-level FPI classification did not materially improve model performance. In contrast, adding C3-FOT qualification in both PV sides increased the C-index to 0.705 and significantly improved model fit (*Δ*LR *χ*^2^ = 11.911; *P* < 0.001). Total gap burden and total VisiTag score also improved model fit, with C-index values of 0.696 and 0.697, respectively (*Δ*LR *χ*^2^ = 8.328, *P* = 0.004; and *Δ*LR *χ*^2^ = 10.627, *P* = 0.031, respectively).

**Table 4:** Incremental model performance for recurrence during follow-up.

Model	Added predictor	LR *χ*²	*Δ*LR *χ*² vs baseline	−2 Log-Likelihood	C-index (95% CI)	*P* for LR test
Model 0	Baseline clinical model	33.130	Ref.	865.864	0.685 (0.627–0.742)	-
Model 1	Model 0 + binary FPI classification	36.145	3.015	862.849	0.687 (0.629–0.744)	0.083
Model 2	Model 0 + three-level FPI classification	36.982	3.853	862.012	0.685 (0.627–0.742)	0.146
Model 3	Model 0 + C3-FOT qualified in both PV sides	45.041	11.911	853.953	0.705 (0.650–0.760)	**<0**.**001**
Model 4	Model 0 + total gap burden	41.458	8.328	857.536	0.696 (0.639–0.754)	**0**.**004**
Model 5	Model 0 + total VisiTag score	43.757	10.627	855.237	0.697 (0.640–0.753)	**0**.**031**

Model 0 (Baseline clinical model included age, sex, BMI, AF type, AF duration, LAD, heart failure, and LVEF).

C-index, concordance index; LR, likelihood ratio; CI, confidence interval; FPI, first-pass isolation; PV, pulmonary vein.

Bold *P* values indicate statistical significance (*P* < 0.05).

For 1-year recurrence prediction, adding FPI status or other procedural metrics to the baseline clinical model yielded modest AUC increases, none of which were significant by DeLong test. C3-FOT qualification in both PV sides showed the largest AUC increase, from 0.696 to 0.728 (*P* = 0.137). Moreover, incorporating C3-FOT qualification in both PV sides significantly improved risk reclassification, with an NRI of 0.456 and an IDI of 0.038 (*P* < 0.05). Total gap burden and total VisiTag score also showed significant IDI values of 0.030 and 0.031, respectively (*P* < 0.05), whereas their NRI values were not significant (Supplementary File S7).

## Discussion

**Figure 5:**
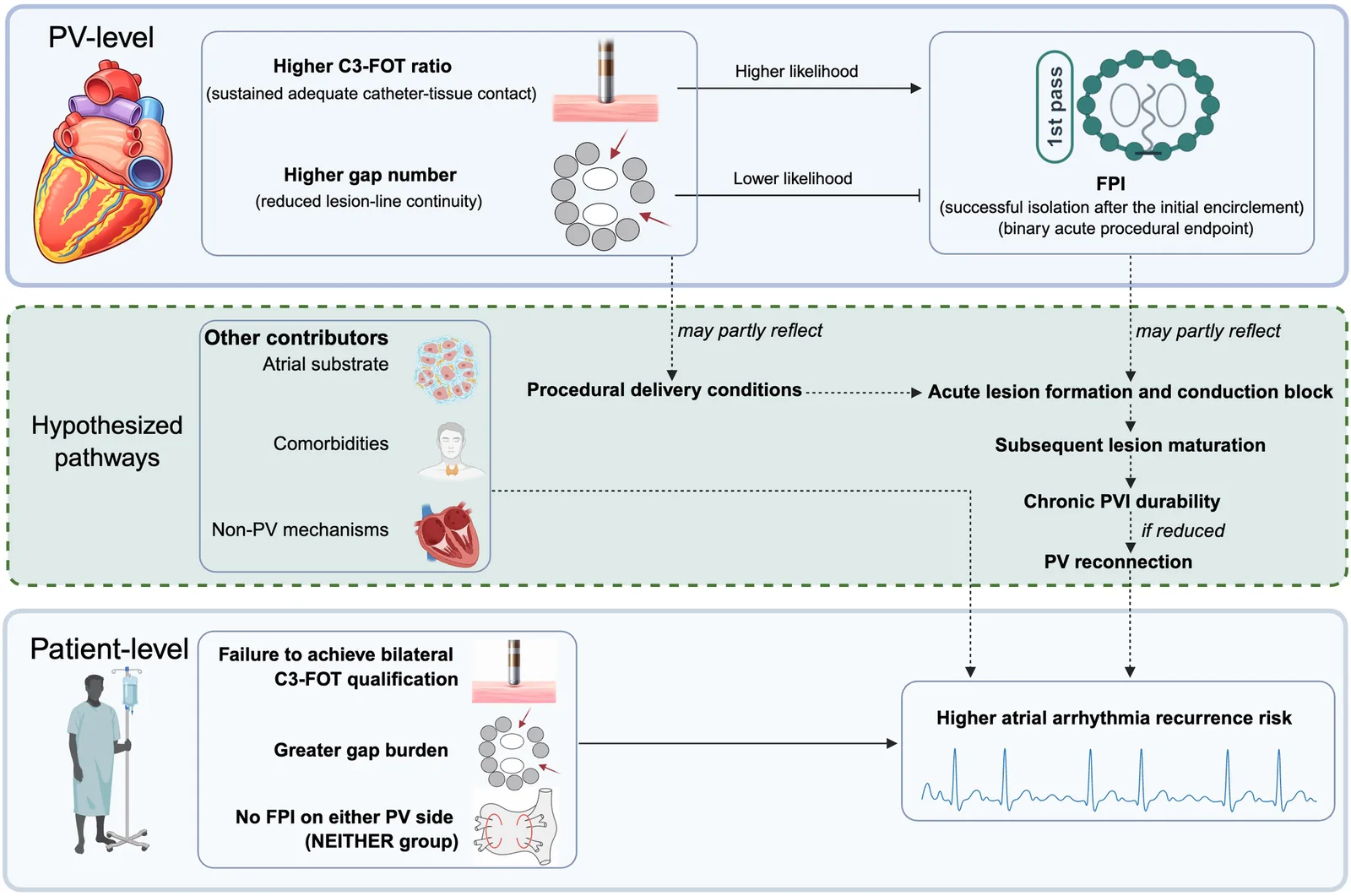
Framework of observed associations and hypothesized pathways linking procedural metrics, first-pass isolation, and atrial arrhythmia recurrence. At the PV level, a higher C3-FOT ratio and a lower gap number were associated with a greater likelihood of FPI. At the patient level, failure to achieve bilateral C3-FOT qualification, total gap burden >2, and no FPI on either PV side (NEITHER group) were each associated with a higher risk of atrial arrhythmia recurrence in separate adjusted models. Solid connectors indicate associations observed in the present study, whereas dashed arrows indicate hypothesized but unmeasured pathways. The C3-FOT ratio was defined as the proportion of lesions within a PV circle with contact force >4 g for more than 30% of the ablation duration. C3-FOT qualification was defined as a ratio ≥75%. FPI, first-pass isolation; PV, pulmonary vein; PVI, pulmonary vein isolation. This figure was created using BioRender.com and is an original figure incorporating BioRender content under a valid Academic Publication License (Agreement No. JT2A0RHPQV).

This study provides clinically relevant insights into the management of AF from the perspective of FPI achievement during PVI. The principal findings were as follows: (1) FPI achievement was closely associated with upstream lesion-delivery metrics, particularly higher C3-FOT ratio and lower gap burden. (2) Absence of FPI in both ipsilateral PVs (NEITHER group) was associated with a higher risk of atrial arrhythmia recurrence after initial RFCA. (3) Although FPI was associated with recurrence, C3-FOT qualification appeared to provide more prognostic information than FPI beyond traditional clinical factors. To synthesize these findings, Figure 5 presents a mechanism-oriented framework distinguishing observed associations from hypothesized but unmeasured pathways. C3-FOT ratio and gap burden represent upstream procedural features related to catheter–tissue contact and lesion-line continuity, whereas FPI is an integrative binary acute electrophysiological endpoint. Recurrence is a downstream multifactorial outcome influenced by procedural and non-procedural factors. Pathways involving lesion maturation, chronic PVI durability, and PV reconnection remain biologically plausible but were not directly evaluated in the present study.

PVI remains the foundation of catheter ablation for AF (3), despite ongoing uncertainty regarding the optimal adjunctive strategy, particularly in persistent AF. Contemporary guidelines and consensus statements have consistently emphasized that durable PVI is central to successful rhythm control. Although adjunctive ablation targeting non-PV triggers or atrial substrates was expected to further improve ablation outcomes, current evidence including several major randomized trials (17–21) has generally shown limited or inconsistent benefit of adjunctive ablation beyond PVI in reducing recurrence. This may be attributed to the challenge in identifying effective non-PV targets (22), and the improved PVI durability enabled by advanced technologies such as AI algorithms and contact force-sensing catheters (23, 24), which may reduce the potential gain from additional ablation. These developments underscore the importance of achieving high-quality and durable PVI. In this context, FPI is clinically meaningful as an immediately observable acute procedural endpoint indicating whether the initial encirclement achieved electrical isolation. As such, it represents an integrative binary electrophysiological result that may reflect lesion continuity, catheter–tissue contact, energy delivery, and anatomical or tissue-related factors (5), but it is not a direct measure of lesion quality and does not by itself establish chronic lesion durability. Achieving FPI may also improve procedural efficiency by reducing the need for further mapping and touch-up ablation, which is supported by our finding that first-pass PVs required shorter PVI and RF times and fewer ablation lesion points. Moreover, recurrence after ablation does not necessarily indicate loss of PV isolation, because recurrences may coexist with durable vein isolation during repeat ablation procedures (24, 25). Accordingly, in cohorts without systematic longitudinal reassessment of PV conduction, FPI should be interpreted as an accessible acute procedural marker rather than as a surrogate for chronically durable PVI.

Consistent with previous studies (6–9), our findings indicated that FPI is associated with more favorable clinical outcomes after RFCA. Patients in the NEITHER group had significantly lower atrial tachyarrhythmia recurrence-free survival. One possible explanation is that FPI after the initial encirclement may reflect a more contiguous initial lesion set. Whether this corresponds to more transmural or durable lesions, however, cannot be determined from the present data. Although touch-up ablation can eliminate residual PV potentials, initial energy delivery to the myocardium may provide a more favorable opportunity to induce durable tissue injury and permanent fibrosis (26). By contrast, when FPI is not achieved, residual conduction and nontransmural lesions may persist. Acute PV wall thickening related to tissue edema has been reported after RF ablation (27), raising the possibility that subsequent RF applications may be less effective in creating durable lesions. Prior imaging studies and reviews suggest that acute conduction block after touch-up ablation may partly reflect reversible edema-related electrical inhibition and therefore may not necessarily indicate durable PVI (28, 29). Nevertheless, the present study assessed acute electrical isolation and clinical recurrence but did not include systematic longitudinal reassessment of PV conduction or histological assessment of lesion formation. Chronic lesion maturation and PV reconnection should therefore be regarded as unmeasured potential mediators rather than mechanisms demonstrated in this cohort. In addition, post-ablation recurrence may reflect non-PV triggers or the underlying atrial substrate even when PV isolation is maintained.

In our study, recurrence-free survival was highest in the BOTH group, intermediate in the EITHER group, and lowest in the NEITHER group. However, the EITHER group did not differ significantly from either the BOTH or NEITHER group in pairwise comparisons, and EITHER group was not significantly associated with recurrence in Cox model using the BOTH group as the reference. Similarly, previous studies identified achievement of FPI in at least one ipsilateral PV circle (IFPI group) as a significant predictor of clinical success, rather than requiring bilateral FPI (7–9). This finding may be attributed to several factors. First, atrial arrhythmia recurrence after ablation is not determined solely by PV lesion quality. Baseline substrate-related factors, including AF type, AF duration, and left atrial remodeling, may also contribute to recurrence (11). This may be particularly relevant in persistent AF, in which progressive atrial remodeling may shift arrhythmia maintenance from PV-trigger dependence toward a more self-sustaining atrial substrate (30). Accordingly, the potential benefit of achieving bilateral FPI over unilateral FPI may be partly offset by residual atrial substrate abnormalities. Second, the arrhythmogenic contribution of PVs may not be symmetric, and left- and right-sided PVs differ in anatomy, catheter stability, myocardial thickness, and susceptibility to reconnection (31, 32). Prior studies have suggested that AF triggers may arise predominantly from one side, particularly the left PVs (33, 34). In this context, the absence of a clear outcome difference between unilateral and bilateral FPI could reflect a predominance of triggers from one PV side in some patients. However, trigger origin and side-specific chronic PV reconnection were not assessed, and all patients ultimately underwent bilateral PVI. In our study, left-sided PVs had a higher FPI success rate than right-sided PVs (71.2% vs. 57.8%, *P* < 0.001), consistent with previous evidence (6, 8, 9, 32). This finding is consistent with the hypothesis that left-sided FPI may have been sufficient to suppress a dominant trigger source in some patients, although this interpretation remains speculative. Moreover, grouping all unilateral FPI patterns into the EITHER category may have introduced mechanistic heterogeneity and diluted its association with recurrence. Nevertheless, these mechanistic interpretations should be considered with caution and warrant further validation. Third, the limited sample size within each subgroup may have constrained the statistical power for pairwise comparisons in the three-level FPI classification. Larger cohorts are needed to determine whether bilateral FPI provides additional prognostic benefit beyond IFPI.

Regarding gap burden, higher gap number was associated with reduced FPI achievement, and first-pass PVs were less likely to contain big gap. Mechanistically, greater gap burden indicates poorer lesion contiguity along the encircling line. Previous studies have also highlighted the importance of ILD for durable PVI, identifying maximal ILD as an independent risk factor for PV reconnection, with maximal ILD < 6 mm (i.e., no gap) linked to improved PVI durability (35) and ILD > 8 mm (i.e., big gap) reported to predict late PV reconnection (36). In addition, a randomized study suggested that targeting a shorter ILD of 3–4 mm improved FPI rates compared with a 5–6 mm target (37). Collectively, these findings support lesion contiguity as an important determinant of acute FPI and suggest a possible association with long-term PVI durability. In our Cox analysis, a total gap count >2 was significantly associated with a higher risk of atrial arrhythmia recurrence, consistent with prior studies (7). Magnetic resonance imaging evidence has shown that lesion volumes may substantially decrease by 90 days after ablation, suggesting that conduction gaps observed acutely could become more prominent as lesions mature (38). Furthermore, recurrence has been associated with the discrepancy between acute ablation coverage and the chronic scar area (39). Thus, acute touch-up ablation may eliminate residual conduction during the procedure, but sites requiring touch-up may remain susceptible to chronic gap formation during lesion maturation, potentially contributing to later PV reconnection and recurrence.

Beyond lesion contiguity, our data indicated that the C3-FOT ratio was another major correlate of FPI achievement, reflecting the temporal and biomechanical quality of catheter–tissue engagement during RF delivery. Although higher mean contact force has been reported to shorten ablation time without necessarily improving outcomes (40), a recent study using a catheter combining contact force sensing with local impedance monitoring showed that higher contact force was associated with an increased likelihood of an adequate local impedance drop, while an adequate local impedance drop predicted first-pass PVI (41). These findings highlight the complementary roles of contact-force information and real-time indicators of tissue response in assessing RF lesion delivery. From this perspective, the C3-FOT ratio should not be interpreted merely as a measure of mean contact force. Rather, a lower C3-FOT ratio may be consistent with micro-instability or transient contact loss during lesion delivery, which could blunt effective resistive heating, attenuate impedance fall, and increase heterogeneity in lesion geometry despite apparently acceptable lesion-index targets. Experimental and clinical studies indicate that contact force, force stability, lesion contiguity, and catheter orientation influence lesion geometry and may affect chronic durability. Greater contact-force variability, catheter drift, or perpendicular contact has been associated with smaller lesions and a higher likelihood of subsequent PV reconnection (42, 43). In our cohort, the linear spline pattern further suggested that higher C3-FOT ratio was progressively associated with a higher likelihood of FPI, rather than only above a discrete threshold. The importance of stable contact may be amplified at the PV antrum, where anisotropic myocardial sleeves, variable wall thickness, complex fiber orientation, and potential epicardial connections create a substrate vulnerable to residual conduction. In such regions, even a short segment of shallow or nonuniform injury may leave viable conducting channels and prevent immediate isolation (44, 45). Consistent with this concept, a previous study (8) reported that touch-up ablation sites after failed FPI frequently clustered at the intervenous carina, a region within the PV antrum that is prone to epicardial muscular bundles connecting the LA and PVs (46). Incomplete endocardial injury in this region could therefore contribute to acute FPI failure, whereas residual epicardial connections might facilitate later electrical reconnection (47), although neither mechanism was directly assessed in our study.

In our cohort, BMI was significantly higher in the NEITHER group, consistent with previous findings suggesting an adverse association between obesity and FPI rates (5). This association may partly reflect more challenging catheter manipulation, reduced respiratory stability, less stable catheter-tissue contact, or altered baseline impedance. Moreover, the observed association between greater atrial dilation and reduced FPI achievement may reflect the influence of atrial remodeling and comorbidities on both extra-PV substrate and lesion delivery (5), although these mechanistic explanations remain speculative. Thus, FPI may be viewed as an integrative acute electrophysiological endpoint reflecting both upstream lesion-delivery conditions and the underlying atrial substrate. These considerations may explain why FPI was clinically informative but added limited incremental prognostic value beyond conventional clinical predictors. Given the observational design and the interrelated nature of these measures, the present study cannot determine whether FPI provides prognostic information independently of upstream procedural and patient-related factors or primarily integrates their combined effects. FPI indicates whether acute electrical isolation is achieved after the initial encirclement, but it neither identifies which component of lesion formation is suboptimal nor establishes chronic PVI durability or the extent of residual lesion vulnerability. Final acute PVI can still be achieved by touch-up ablation despite FPI failure. By contrast, failure to achieve C3-FOT qualification on both PV sides and greater total gap burden yielded larger improvements in model discrimination and model fit, with C3-FOT showing the most consistent enhancement in model performance and risk reclassification. This may reflect the more granular procedural information provided by gap burden and C3-FOT, which characterize lesion-line continuity and the temporal maintenance of catheter–tissue contact, respectively, and may not be fully captured by FPI status. Nevertheless, the overall incremental prognostic gain remained modest, supporting their value as complementary tools for procedural quality assessment and risk stratification, rather than as a basis for reliable recurrence prediction models.

In the present study, gaps were most frequently observed at the right bottom and left anterior segments. A previous study reported more gaps at the right posterior and left anterior segments, and proposed that local atrial contraction and passive cardiac motion may reduce catheter stability in these regions (7). The discrepancy in right-sided distribution may reflect differences in sheath support, catheter approach and procedural workflow. Notably, both our findings and prior evidence consistently showed frequent gap formation at the left anterior segment. In our study, the clustering of C3-FOT deficiency and touch-up sites in this area may be consistent with reduced local catheter stability, less favorable approach vectors and contact-angle constraints. These observations raise the possibility that very-high-power short-duration (90-W/4-second) ablation may partly mitigate the burden of catheter instability by shortening the required duration of stable contact. From a biophysical perspective, this strategy has been proposed to enhance lesion contiguity and transmurality by producing a greater proportion of resistive heating within a brief application window (48). However, this theoretical advantage should be interpreted cautiously. A previous study reported a numerically lower, but nonsignificant FPI rate in the 90-W group compared with controls (49). Moreover, 90-W/4-s lesions are relatively shallow and small, and may require tighter interlesion spacing (50). In this context, insufficient contact during such a brief application could further compromise lesion depth and contiguity.

Beyond modifications of RF energy delivery, pulsed field ablation (PFA) has emerged as an alternative nonthermal modality for PVI. In the PFA setting, FPI refers to successful electrical isolation of the targeted PV using only the initially planned sequence of PFA applications, without additional touch-up applications. In the PFA dose study, a simplified protocol using four applications per PV achieved an FPI rate similar to that of the standard protocol using eight applications per PV (95% vs. 95%), while reducing procedure duration and left atrial dwell time (51). These findings suggest that FPI may also serve as a practical acute procedural endpoint during PFA. However, whether FPI has the same procedural determinants and clinical implications in PFA as in RF ablation remains to be clarified.

From a broader clinical perspective, AF may be precipitated or sustained by modifiable, potentially reversible, or underlying disease-related factors. Hypertension is an important modifiable risk factor for AF (3), whereas hyperthyroidism may precipitate AF or atrial flutter (52). Long-term participation in endurance sports has been associated with AF in selected populations (53), alcohol consumption is a modifiable risk factor associated with AF occurrence and recurrence (3), and smoking is associated with incident AF (54). In selected patients, evaluation for inherited electrical or structural myocardial disease may also be relevant. Identification of a channelopathy such as Brugada syndrome may warrant broader arrhythmic risk assessment and individualized management (55), whereas cardiomyopathy may represent an underlying substrate associated with AF and adverse clinical outcomes (56). Accordingly, identifying and, where appropriate, managing these factors may reduce future AF burden regardless of the ablation strategy and, in selected patients, may prompt reassessment of the indication for ablation.

Several limitations should be acknowledged. First, owing to the retrospective observational design, selection bias and residual confounding could not be fully excluded. Second, although AIFV-derived metrics, including the C3-FOT ratio, were quantitatively assessed, the thresholds used in this study have not been externally validated, which may limit their generalizability and warrants further validation. Third, rhythm follow-up was based on scheduled 24-h Holter monitoring and symptom-triggered electrocardiography rather than continuous monitoring. Consequently, asymptomatic, short-duration, or intermittent episodes occurring outside the scheduled monitoring windows may have been missed, potentially leading to underestimation of the recurrence rate and misclassification of some patients as recurrence-free. Moreover, without systematic repeat electrophysiological mapping, chronic PV conduction status was not directly assessed in our study, and we could not determine whether FPI and AIFV-derived lesion metrics reflected durable PVI or whether PV reconnection contributed to the association between FPI and clinical outcomes. Although these approaches were beyond the scope of the present retrospective study, waiting-period reassessment can identify spontaneous acute PV reconnection, while ATP/adenosine challenge can unmask dormant conduction (8). Longer-term PVI durability may be assessed by repeat electrophysiological mapping in selected patients (6, 10), with cardiac MRI providing complementary structural characterization of ablation scar and lesion maturation (38, 39). Finally, this was a single-center study, and the prognostic relevance of FPI should be confirmed in larger prospective multicenter cohorts.

## Conclusion

As an acute procedural endpoint, FPI was associated with favorable ablation outcomes after RFCA for AF. IFPI was independently associated with a lower risk of atrial arrhythmia recurrence. FPI achievement was associated with procedural characteristics, including a lower gap burden and a higher C3-FOT ratio. However, the incremental prognostic value of FPI beyond conventional clinical predictors was limited. Procedural metrics, particularly C3-FOT qualification, may provide more granular information for procedural quality assessment and post-ablation risk stratification. Further prospective multicenter studies are warranted to validate these observations.

## Data Availability

The raw data supporting the conclusions of this article will be made available by the authors, without undue reservation.
