# Supplementary material for: First-pass isolation and procedural metrics associated with catheter ablation outcomes for atrial fibrillation: a retrospective cohort study
Source: Front Cardiovasc Med. 2026 Aug 25;13:1900303. doi: 10.3389/fcvm.2026.1900303 (PMC13550968; doi:10.3389/fcvm.2026.1900303)
Supplement: Supplementary file 1 [file Datasheet1.docx]

| Supplementary file 1. Scoring criteria of the AIFV system | |
| --- | --- |
| Visitag score | Criteria |
| V6 | Persistent atrial fibrillation, both PV circles scored V5, procedural time for both circles ≤ 60 minutes, number of ablation lines ≥ 2 |
| V5 | Integrated break point ratio ≤ 25.0%, C3 FOT (4g,30%) ≥75%, SURPOINT(Top4) ≥ 80%, Gap ≤ 2, bigGap = 0 |
| V4 | Integrated break point ratio ≤ 40.0%, C3 FOT (4g,30%) ≥60%, SURPOINT(Top4) ≥ 70%, Gap ≤ 2, bigGap = 0 |
| V3 | Integrated break point ratio ≤ 50.0%, Gap ≤ 5 |
| V2 | Other circumstances |

Supplementary file 2. Schematic illustration of pulmonary vein segmentation

| Supplementary file 3. Baseline and procedural characteristics by FPI status | | | |
| --- | --- | --- | --- |
| Characteristics | IFPI group (n=253) | NEITHER group (n=60) | *P* value |
| Demographic and clinical characteristics | | | |
| Age, years | 62.0 (55.0-70.0) | 60.0 (54.3-70.0) | 0.832 |
| Male, n (%) | 147 (58.1) | 36 (60.0) | 0.789 |
| BMI | 24.6 (22.4-26.7) | 25.9 (23.6-28.1) | **0.029** |
| Smoke, n (%) | 79 (31.2) | 15 (25.0) | 0.344 |
| Drink, n (%) | 76 (30.0) | 14 (23.3) | 0.302 |
| Paroxysmal AF, n (%) | 154 (60.9) | 34 (56.7) | 0.550 |
| Presence of other arrhythmias, n (%) | 63 (24.9) | 15 (25.0) | 0.987 |
| Antiarrhythmic drug, n (%) | 101 (39.9) | 27 (45.0) | 0.472 |
| CHA2DS2‐VASc score | 2.0 (1.0-3.0) | 2.0 (1.0-3.0) | 0.558 |
| HAS-BLED score | 1.0 (0.0-2.0) | 1.0 (0.0-2.0) | 0.685 |
| AF duration, months | 12.0 (3.0-25.0) | 12.0 (3.0-36.0) | 0.236 |
| Comorbidities, n (%) | | | |
| Heart failure | 33 (13.0) | 10 (16.7) | 0.464 |
| Hypertension | 108 (42.7) | 25 (41.7) | 0.886 |
| Diabetes mellitus | 40 (15.8) | 10 (16.7) | 0.871 |
| CAD | 35 (13.8) | 7 (11.7) | 0.658 |
| History of stroke | 24 (9.5) | 9 (15.0) | 0.211 |
| Thyroid disease | 15 (5.9) | 1 (1.7) | 0.178 |
| COPD | 11 (4.3) | 4 (6.7) | 0.450 |
| Echocardiographic data | | | |
| LAD, mm | 39.0 (35.0-42.0) | 39.0 (37.0-45.8) | 0.054 |
| LVD, mm | 48.0 (45.0-51.0) | 47.5 (44.0-52.0) | 0.543 |
| LVEF, % | 64.0 (61.0-69.0) | 63.5 (57.0-70.8) | 0.185 |
| Laboratory data | | | |
| Hb, g/l | 141.0 (131.0-152.5) | 140.0 (134.0-151.8) | 0.794 |
| Cre, umol/l | 80.0 (68.0-92.0) | 82.5 (66.3-93.3) | 0.872 |
| FIB, g/l | 2.62 (2.30-3.06) | 2.59 (2.26-3.10) | 0.568 |
| proBNP, pg/ml | 365.0 (85.0-863.5) | 489.5 (63.8-1167.5) | 0.623 |
| Procedural details | | | |
| PVI plus, n (%) | 85 (33.6) | 18 (30.0) | 0.594 |
| Total procedure time, min | 60.1 (45.6-80.9) | 70.9 (56.7-86.0) | **0.002** |
| Gap of PVI circle | 1.0 (0.0-1.0) | 1.0 (0.0-3.0) | **< 0.001** |
| Big gap, n (%) | 22 (8.7) | 12 (20.0) | **0.011** |
| C3-FOT qualified in left PV side, n (%) | 233 (92.1) | 40 (66.7) | **< 0.001** |
| C3-FOT qualified in right PV side, n (%) | 219 (86.6) | 34 (56.7) | **< 0.001** |
| Total VisiTag score, n (%) |  |  |  |
| V2 | 1 (0.4) | 3 (5.0) | **< 0.001** |
| V3 | 32 (12.6) | 21 (35.0) |  |
| V4 | 65 (25.7) | 23 (38.3) |  |
| V5 | 124 (49.0) | 12 (20.0) |  |
| V6 | 31 (12.3) | 1 (1.7) |  |
| AF, atrial fibrillation; FPI, first-pass isolation; CAD, coronary artery disease; COPD, chronic obstructive pulmonary disease; proBNP, pro-brain natriuretic peptide; Cre, creatinine; FIB, fibrinogen; Hb, hemoglobin; LAD, left atrial diameter; LVD, left ventricular diameter; LVEF, left ventricular ejection fraction; PV, pulmonary vein; PVI, pulmonary vein isolation. | | | |

| Supplementary file 4. PV-level procedural characteristics | | | |
| --- | --- | --- | --- |
| Procedural details | First-pass PVs (n=404) | Non-first-pass PVs (n=222) | *P* value |
| Right-sided PV, n (%) | 181 (44.8) | 132 (59.5) | **< 0.001** |
| Touch-up ablation time, min | - | 7.0 (3.0-10.0) | - |
| Break point ratio, % | 0 (0.0-9.3) | 0.9 (0.0-11.3) | **0.006** |
| Gap | 0 (0.0-1.0) | 0.0 (0.0-2.0) | **< 0.001** |
| Big gap, n (%) | 16 (4.0) | 27 (12.2) | **< 0.001** |
| C3-FOT ratio, % | 91.8 (85.3-97.1) | 78.8 (70.9-88.5) | **< 0.001** |
| C3-FOT qualified, n (%) | 385 (95.3) | 141 (63.5) | **< 0.001** |
| SURPOINT (Top4) ratio, % | 100 (95.7-100) | 97.5 (93.5-100.0) | **0.001** |
| Total PVI time, min | 19.6 (15.6-24.1) | 22.4 (17.5-27.2) | **< 0.001** |
| RF time, min | 7.9 (6.9-9.1) | 8.7 (7.5-10.2) | **< 0.001** |
| RF time ratio, % | 41.5(35.2-48.9) | 40.4 (34.0-46.6) | 0.165 |
| Ablation lesion points | 38.0 (33.0-42.0) | 41.0 (36.0-48.0) | **< 0.001** |
| VisiTag score, n (%) |  |  |  |
| V2 | 0 (0) | 5 (2.3) | **< 0.001** |
| V3 | 22 (5.4) | 49 (22.1) |  |
| V4 | 48 (11.9) | 72 (32.4) |  |
| V5 | 334 (82.7) | 96 (43.2) |  |
| PV, pulmonary vein; PVI, pulmonary vein isolation; RF, radiofrequency. | | | |

Supplementary file 5. Multivariable RCS curve for C3-FOT ratio in relation to FPI

| Supplementary file 6. Cox proportional hazards models for recurrence according to procedural metrics | | | | | | | |
| --- | --- | --- | --- | --- | --- | --- | --- |
|  | Model 1 (Crude) | | |  | Model 2 (Adjusted) | | |
|  | HR | 95%CI | *P* |  | HR | 95%CI | *P* |
| C3-FOT qualified | | | | | | | |
| Both ipsilateral PVs | Ref. |  |  |  | Ref. |  |  |
| Eihter/no ipsilateral PV | 2.294 | 1.474-3.569 | **<0.001** |  | 2.315 | 1.456-3.681 | **<0.001** |
| Total gap burden | | | | | | | |
| 0-2 | Ref. |  |  |  | Ref. |  |  |
| >2 | 2.350 | 1.458-3.787 | **<0.001** |  | 2.129 | 1.286-3.524 | **0.003** |
| Big gap | | | | | | | |
| No | Ref. |  |  |  | Ref. |  |  |
| Yes | 1.775 | 0.998-3.158 | 0.051 |  | 1.630 | 0.898-2.958 | 0.108 |
| Total VisiTag score | | | | | | | |
| V5 | Ref. |  |  |  | Ref. |  |  |
| V2 | 13.944 | 4.739-41.027 | **<0.001** |  | 6.425 | 1.907-21.645 | **0.003** |
| V3 | 2.367 | 1.299-4.310 | **0.005** |  | 1.862 | 0.965-3.591 | 0.064 |
| V4 | 1.754 | 0.995-3.090 | 0.052 |  | 1.442 | 0.789-2.635 | 0.234 |
| V6 | 1.816 | 0.840-3.925 | 0.129 |  | 0.898 | 0.365-2.212 | 0.815 |
| HR, hazard ratio; CI, confidence interval; PV, pulmonary vein  Model 1: unadjusted.  Model 2: adjusted for age, sex, BMI, AF type, AF duration, LAD, PVI plus, heart failure, hypertension, diabetes, CAD, history of stroke. | | | | | | | |

| Supplementary file 7. Discrimination and reclassification analyses for 1-year recurrence | | | | | | | | | |  |
| --- | --- | --- | --- | --- | --- | --- | --- | --- | --- | --- |
|  |  | AUC |  |  | NRI | |  | IDI | | |
| Model | Added predictor | Index | *P* for DeLong test |  | Index | *P*-value |  | Index | *P*-value | |
| Model 0 | Baseline clinical model | 0.696 | - |  | - | - |  | - | - | |
| Model 1 | Model 0 + binary FPI | 0.704 | 0.585 |  | 0.306 | ≥0.05 |  | 0.014 | ≥0.05 | |
| Model 2 | Model 0 + three-level FPI achievement | 0.705 | 0.571 |  | 0.102 | ≥0.05 |  | 0.018 | ≥0.05 | |
| Model 3 | Model 0 + C3-FOT qualified in both PV sides | 0.728 | 0.137 |  | 0.456 | **<0.05** |  | 0.038 | **<0.05** | |
| Model 4 | Model 0 + total gap burden | 0.708 | 0.455 |  | 0.050 | ≥0.05 |  | 0.030 | **<0.05** | |
| Model 5 | Model 0 + total VisiTag score | 0.713 | 0.280 |  | 0.179 | ≥0.05 |  | 0.031 | **<0.05** | |
| Model 0 (Baseline clinical model included age, sex, BMI, AF type, AF duration, LAD, heart failure, and LVEF).  AUC, area under the curve; FPI, first-pass isolation; IDI, integrated discrimination improvement; NRI, net reclassification improvement; PV, pulmonary vein. | | | | | | | | | |  |
